# Quarantine-associated viral pathogens in non-human primates: pathobiology, diagnostic surveillance, control strategies, and one health implications

**DOI:** 10.3389/fvets.2026.1933886

**Published:** 2026-09-22

**Authors:** Gyu-Seo Bae, Eunsu Jeon, Joonwon Mo, Ju-Yeon Lee, Ja Rang Lee, Jung Joo Hong, Dong-Ho Lee, Bon-Sang Koo

**Affiliations:** 1 National Primate Research Center, Korea Research Institute of Bioscience and Biotechnology, Cheongju, Republic of Korea; 2 Primate Resources Center, Korea Research Institute of Bioscience and Biotechnology (KRIBB), Jeongeup, Republic of Korea; 3 KRIBB School of Bioscience, Korea University of Science and Technology, Daejeon, Republic of Korea; 4 KRIBB School of Advanced Bioconvergence, Korea University of Science and Technology (UST), Daejeon, Republic of Korea

**Keywords:** B virus, non-human primate, one health, quarantine, virus

## Abstract

Recent outbreaks of emerging infectious diseases, along with advances in neurological and other biomedical research, have increased the use of non-human primates (NHPs) as preclinical models. Their close phylogenetic and physiological similarities to humans make them valuable for translational research, especially during public health emergencies. However, NHPs can harbor a wide range of viral pathogens, some of which pose zoonotic risks and may compromise animal health and experimental reproducibility. Therefore, quarantine procedures, pathogen surveillance, and biosecurity programs are essential for the safe and reliable use of NHPs in research. Despite this need, comprehensive reviews linking national quarantine policies with the biological and epidemiological characteristics of major viral pathogens and their relevance to disease surveillance and control remain limited. This review compares national quarantine frameworks for NHPs, examines pathogen-specific features, and discusses their implications for quarantine and surveillance. We emphasize the need for an integrated, risk-based approach incorporating longitudinal microbiological monitoring, multimodal diagnostic assessment, facility-level response procedures, occupational health preparedness, and internationally harmonized quarantine standards.

## 1 Introduction

Zoonotic infectious diseases are increasingly emerging worldwide, driven by multiple factors, including increased global travel and trade, climate change, urbanization, and land-use change (1). In recent years, viruses with pandemic potential, such as severe acute respiratory syndrome coronavirus 2 (SARS-CoV-2) and highly pathogenic avian influenza viruses (HPAIVs), have posed significant global public health threats. In response to these emerging infectious diseases, various preclinical animal models have been widely used in infectious disease researches, including mice, hamsters, ferrets, guinea pigs, and non-human primates (NHPs) (2). Because effective therapeutics and vaccines may not be readily available for newly emerging viral diseases, preclinical studies using animal models play an important role in the development and evaluation of novel countermeasures.

NHP models are more anatomically, physiologically, immunologically, and genetically similar to humans than other commonly used preclinical animal models. For this reason, NHPs have often been used as important preclinical models to investigate the pathogenesis of infections, caused by Ebola virus, SARS-CoV-2, and H5N1 HPAIVs and to support the development of vaccines and therapeutics against these viruses (3–6). For example, mRNA vaccines targeting SARS-CoV-2 have been evaluated for efficacy and safety using NHP models. In addition, ZMapp, a monoclonal antibody-based therapy against Ebola virus, demonstrated efficacy in treating Ebola virus infection in NHP models (7). These findings from NHP studies provided evidence supporting the clinical translation and emergency use of these interventions during outbreaks under regulatory and ethical frameworks such as the WHO MEURI guidelines (8). Consequently, the use of NHP models has increased significantly in recent years, resulting in a shortage of available NHPs (9, 10).

NHPs have been reported globally to harbor various pathogens that can cause latent or subclinical infections, including B virus (*Cercopithecine herpesvirus* 1), simian immunodeficiency virus (SIV), simian T cell lymphotropic/leukemia virus (STLV), simian type D retrovirus (SRV), and *Mycobacterium* (*M*) *tuberculosis*, all of which should be absent from specific pathogen-free (SPF) colonies (11–13) (Figure 1). Also, simian polyomaviruses, including simian virus 40 (SV40), have been detected macaque species (14). Therefore, latent or subclinical infections in clinically healthy NHPs may compromise the reproducibility of experimental outcomes and pose a risk of zoonotic transmission to laboratory personnel. Potentially lethal zoonotic viruses, including rabies virus, Marburg virus, Ebola virus, monkeypox virus, and yellow fever virus, have been reported in NHP species (15).

**Figure 1:**
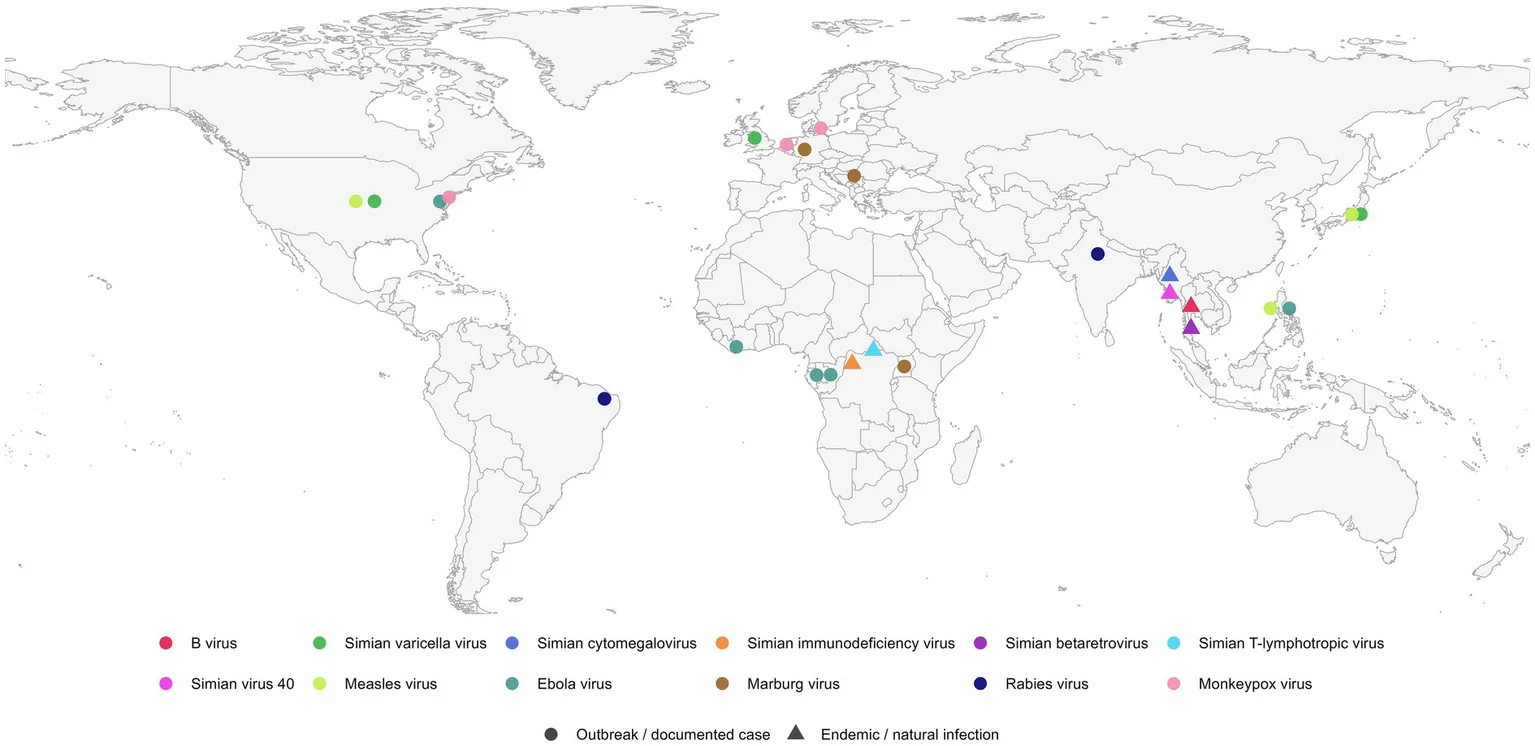
Global distribution of documented viral infections and outbreaks in non-human primates. Geographical locations of documented NHP viral infections and outbreaks reported in the literature; circles, documented natural or captive infections; triangles, reported outbreaks; different colors, individual viruses; locations based on published reports, not necessarily representing the complete geographical distribution or prevalence of each virus. Detailed information on documented infections and outbreaks, including corresponding references, Supplementary Table S1 (127, 162, 179, 182, 198–210).

Experimental NHPs are typically outbred and exhibit greater genetic diversity than many inbred preclinical animal models (16). This heterogeneity may lead to individual variation in susceptibility to infectious agents. Outdoor housing and husbandry practices can further hinder the establishment and maintenance of SPF colonies (17). Therefore, appropriate quarantine procedures are essential for NHPs. This review provides an overview of national quarantine protocols and major viral pathogens relevant to NHPs. We focus on pathogen biology and epidemiology, diagnostic approaches, and interventions, and consider their implications for risk-based quarantine, surveillance, and infection-control strategies. We also highlight the need for coordinated facility-level responses, occupational health preparedness, and internationally harmonized quarantine standards from a One Health perspective.

## 2 International quarantine policies and virus surveillance in NHPs facilities

### 2.1 Republic of Korea

In the Republic of Korea, the importation of NHPs is regulated under the Act on the Prevention of Contagious Animal Diseases, and these species are classified as designated quarantine animals subject to mandatory veterinary inspection upon importation. Experimental macaques, primarily cynomolgus (*Macaca fascicularis*) and rhesus (*Macaca mulatta*) macaques, are predominantly imported from Asian countries, including China, Vietnam, and Cambodia (10, 18). Imported NHPs must undergo at least 30 days of pre-export quarantine under the supervision of a government-designated veterinarian in the exporting country, followed by an additional 30-day quarantine at a designated national animal quarantine facility in Korea prior to release for research use. Specific sanitary import requirements are defined in the national import health regulations for NHP species (19). These regulations require that the exporting country be free from Ebola virus and Marburg virus diseases. The breeding facility of origin must have no evidence of clinical, serological, or pathological occurrence of measles, hepatitis A, hepatitis B, monkeypox, or tuberculosis (*M. tuberculosis* and *M. bovis*) for at least 24 months prior to export. During the mandatory pre-export quarantine period, NHPs must be confirmed negative for tuberculosis, internal and external parasites, and enteric bacterial pathogens, including *Salmonella* spp., *Shigella* spp., and *Yersinia* spp. Tuberculosis screening must include at least one tuberculin skin test (TST) conducted within one week after the start of quarantine, while hepatitis B virus testing is additionally required for gibbons and great apes.

Beyond these national regulatory mandates, research institutions often conduct additional microbiological screening. However, the pathogens targeted for screening vary according to facility policies, research objectives, and biosafety requirements. Additional screening may include testing for B virus, SIV, STLV, SRV, and other facility-specific pathogens.

### 2.2 United states

In the United States, the importation of NHPs is regulated by the Centers for Disease Control and Prevention (CDC) under the Public Health Service Act (20). All imported NHPs must undergo quarantine for a minimum of 31 days in a CDC-registered facility prior to release. At the federal regulatory level, tuberculosis surveillance via serial TST is the only routine laboratory test universally required during quarantine. Each NHP must receive at least three TSTs administered on the eyelid using mammalian old tuberculin (MOT), at intervals of at least 2 weeks. Reactions must be evaluated at 24, 48, and 72 h after each test. Routine laboratory screening for other diseases is not uniformly required, although additional diagnostic testing may be required under specific clinical circumstances, including filovirus testing in Old World NHPs exhibiting clinical signs suggestive of filovirus disease.

Beyond federal requirements, individual research institutions may implement additional quarantine measures based on their own policies. For example, the National Institutes of Health (NIH) requires documentation of a negative TST within 30 days prior to shipment (21). The standard quarantine period is 90 days for recently imported NHPs and 60 days for animals originating from long-established domestic colonies. During quarantine, TSTs using MOT are administered on alternate eyelids at two-week intervals and evaluated at 24, 48, and 72 h. Depending on the species, three to four consecutive negative results are required before release from quarantine. NIH also requires species-specific viral screening within the first 35 days of quarantine for selected simian retroviruses (e.g., SIV, SRV, STLV), herpesviruses (including B virus in macaques), and measles virus, depending on species.

### 2.3 European union

Within the European Union (EU), the movement and importation of live animals, including NHPs, are governed by EU animal health legislation (22). The EU framework requires animal health certification and movement control but does not prescribe a uniform quarantine period or standardized laboratory testing for NHPs. These measures are therefore determined by individual countries in EU. In the United Kingdom, national legislation requires imported NHPs to undergo a four-month quarantine in a government-approved quarantine facility prior to release (23). In France, the importation of NHPs is regulated under national veterinary legislation (24). Unlike the United Kingdom, France does not prescribe a uniform quarantine period or standardized laboratory testing for imported NHPs.

In the EU, routine health surveillance programs for NHPs are largely guided by the recommendations of the Federation of European Laboratory Animal Science Associations (FELASA). These recommendations provide a harmonized framework for detecting and controlling endemic, opportunistic, and newly introduced agents that may cause zoonotic or anthroponotic disease or interfere with biomedical research outcomes (25).

According to the FELASA recommendations, health monitoring programs should be adapted to the species, colony status, and the specific requirements of breeding or user facilities. For macaques, including *Macaca mulatta* and *Macaca fascicularis*, routine viral surveillance includes B virus, SIV, SRV, and STLV. For New World primates such as the common marmoset (*Callithrix jacchus*), surveillance programs may additionally include species-specific viral pathogens according to colony history and institutional risk assessment (25).

FELASA further emphasizes the importance of validated diagnostic methodologies, including serological and molecular assays, as well as standardized health reporting and certification systems. Recommended testing frequencies vary according to species, pathogen profile, colony health status, and institutional management strategy. Health monitoring is recommended to be performed by appropriately qualified laboratories using validated procedures to ensure the reliability and comparability of diagnostic results (25).

### 2.4 Japan

In Japan, the importation of NHPs is administered by the Ministry of Agriculture, Forestry and Fisheries through the Animal Quarantine Service (AQS) under applicable ministerial ordinances and animal health requirements (26–28). Importation is restricted to designated exporting countries and requires advance notification and official veterinary health certification. A minimum 30-day pre-export quarantine at facilities designated by the Minister of Agriculture, Forestry and Fisheries is required prior to shipment.

Upon arrival in Japan, imported NHPs undergo a minimum 30-day detention inspection conducted by the AQS. During this period, animals are monitored for high-consequence zoonotic pathogens, particularly viral hemorrhagic fevers such as Ebola and Marburg virus diseases, in accordance with national sanitary requirements. Following completion of national quarantine, research institutions typically implement additional health screening and microbiological monitoring according to institutional biosafety policies and SPF colony management standards (29). National regulatory requirements for quarantine duration and pathogen screening of imported NHPs in selected regions are summarized in Table 1.

**Table 1:** Quarantine duration and pathogen screening requirements for imported nonhuman primates under national regulations.

Region/Country	Quarantine duration	Viral screening	Bacterial screening	Parasitic screening	Tuberculosis testing
South Korea	≥30 days pre-export + 30 days post-entry	Ebola, Marburg (country disease-free requirement)	*Salmonella, Shigella, Yersinia* (pre-export)	Internal and external parasites (pre-export)	≥1 TST (pre-export)
		Measles, hepatitis A/B, monkeypox (facility disease-free requirement)			
United States	≥31 days post-entry	Not routinely mandated; filovirus testing if clinically indicated	Not routinely mandated	Not routinely mandated	≥3 TSTs
European Union	Not specified	Not specified	Not specified	Not specified	Not specified
United Kingdom	~4 months quarantine	Not specified	Not specified	Not specified	Not specified
Japan	≥30 days pre-export + ≥ 30 days post-entry	Ebola, Marburg (country disease-free requirement)	Not specified	Not specified	Not specified

“Not specified” indicates that the relevant testing requirement is not explicitly defined in the national legislation reviewed. In South Korea, most pathogen screening is conducted during the mandatory pre-export quarantine in the exporting country. TST, tuberculin skin test; MOT, mammalian old tuberculin.

## 3 Characteristics of major viral pathogens

This report primarily focuses on major viral pathogens of relevance to NHP quarantine and health surveillance in Korea, the United States, Japan, and European countries, particularly those that generally cause subclinical infection or no apparent clinical signs. It also discusses viral diseases associated with severe or fatal outcomes, some of which are subject to quarantine measures in certain countries.

### 3.1 Simian herpesviruses

Herpesviruses are enveloped viruses with an icosahedral capsid, a proteinaceous tegument, and a linear double-stranded DNA genome. A major characteristic of herpesviruses is their ability to establish lifelong latent infections with subsequent reactivation. Major herpesviruses of concern in NHPs include herpes B virus (B virus), simian varicella virus (SVV), and simian cytomegalovirus (CMV).

#### 3.1.1 Herpes B virus (B virus)

##### 3.1.1.1 Etiology

B virus, *Macacine alphaherpesvirus* 1, is an alphaherpesvirus belonging to the genus *Simplexvirus* within the family *Herpesviridae*. It is considered the principal zoonotic herpesvirus of macaques (30, 31). Like other alphaherpesviruses, B virus establishes lifelong latency following primary infection and undergoes intermittent reactivation with episodic viral shedding. The viral genome is approximately 155–160 kb in length and shares a broadly conserved genomic organization with human herpes simplex viruses (HSVs), including HSV-1 and HSV-2, although notable genetic differences are present. Molecular studies have demonstrated that B virus isolates cluster by macaque host species, suggesting host-associated genotypes and probable virus–host co-speciation (32). More recent sequence analyses, including studies of glycoprotein D divergence among clinical and laboratory isolates, have also demonstrated genetic heterogeneity among B virus strains and identified conserved genomic regions that may serve as targets for diagnostic assays (33). Comparative genomic and immunological analyses suggest that B virus differs from HSV in its neuroinvasion properties and modulation of host immune responses. These differences may contribute to the severe central nervous system manifestations in humans, whereas infection in macaques is typically mild (34).

##### 3.1.1.2 Host

B virus is enzootic in Asian macaques (*Macaca* spp.), including rhesus, cynomolgus, Japanese, pig-tailed, bonnet, and other macaque species (30). Infection in macaques is typically mild or subclinical but lifelong. Humans are accidental hosts, and although zoonotic infection is rare, symptomatic infection can progress to severe, often fatal neurologic disease, particularly when the central nervous system is involved. Because macaques are widely used in biomedical research and may also be encountered in zoological collections, tourism settings, and private ownership, human exposure may occur through occupational or other close contact with macaques (30). Cross-species transmission to non-macaque primates has also been reported and may result in severe disease, indicating a potential risk when macaques are housed with other primate species (30).

##### 3.1.1.3 Clinical signs and pathogenesis

In macaques, primary infection is often asymptomatic. When clinical disease occurs, it typically manifests as transient vesicular or ulcerative lesions of the oral mucosa. Disseminated disease in macaques is rare but can involve necrotizing lesions in the liver, lung, or central nervous system (31, 35). In contrast, human infection can progress to rapid neuroinvasion and ascending encephalomyelitis. Early symptoms may include localized vesicular lesions, paresthesia, fever, and lymphadenopathy, followed by progressive neurologic dysfunction. Before antiviral therapy became available, the case fatality rate was reported to exceed 70% (30). Neurologic sequelae are common among survivors. Early diagnosis and initiation of antiviral therapy may reduce mortality and neurologic complications.

##### 3.1.1.4 Transmission route

In macaques, transmission occurs primarily through mucosal contact (oral, genital or conjunctival), bites, or exposure to infectious secretions. Although vertical or perinatal transmission cannot be entirely excluded, it is not considered a major route of transmission in macaque populations. Viral shedding is intermittent and often occurs without clinical signs following reactivation from latency in sensory ganglia (30). Reactivation and viral shedding may occur more frequently during periods of stress, immunosuppression, or environmental change (30, 31, 35). In humans, most documented infections have occurred following macaque bites, scratches, or mucosal exposure to saliva or other secretions. Occupational infection has also been reported following exposure to macaque cell cultures or central nervous system tissues (30).

##### 3.1.1.5 Epidemiology

Seroprevalence in adult macaques in conventional colonies and wild populations commonly ranges from approximately 70% to nearly 100%, and increases with age (30, 36, 37). In contrast, active viral shedding is uncommon and occurs in only a small proportion of animals at a given time. Although human infection is rare relative to the frequency of human–macaque contact, approximately 50 human cases have been documented since B virus was first identified in 1932, including fatal occupational infections (30). Despite the rarity of human infection, its potentially severe or fatal outcome requires strict preventive measures for individuals working with macaques.

#### 3.1.2 Diagnostics, vaccines, and therapeutics

##### 3.1.2.1 Diagnostics

Diagnosis of B virus infection is complicated by lifelong latency, intermittent reactivation, and antigenic cross-reactivity with related alphaherpesviruses. Serologic screening using ELISA, with Western blot as a confirmatory test has been widely used for colony surveillance and exposure assessment. However, cross-reactivity with human HSV antibodies can reduce specificity in human samples, and recently infected animals may test negative before seroconversion (30, 31).

Molecular detection using quantitative real-time PCR (qPCR) provides greater analytical sensitivity and specificity than conventional PCR and viral culture. Early TaqMan-based assays targeting conserved regions such as glycoprotein genes were developed for clinical diagnosis and surveillance (38). qPCR assays have also been developed for field and wildlife surveillance, including the testing of oral swabs collected from free-ranging macaques (39). Despite the high sensitivity of qPCR, negative PCR results from asymptomatic animals do not exclude infection because viral shedding is episodic. Therefore, a single negative PCR result cannot rule out B virus infection. Whole-genome sequencing (WGS) allows high-resolution differentiation of macaque-associated B virus strains (33), although it is not currently used for routine diagnostic screening.

##### 3.1.2.2 Vaccination and therapeutics

There is currently no licensed vaccine for B virus in humans or macaques. Experimental vaccine approaches have been investigated, but none have progressed to routine use (31). Immediate wound cleansing (for at least 15 min using detergent and running water) is recommended following potential exposure and may reduce the risk of infection (40). Antiviral therapy with valacyclovir or acyclovir is recommended for high-risk exposures, and intravenous acyclovir or ganciclovir is indicated for suspected or confirmed CNS involvement (31, 41). Fatal infection has not been reported in individuals who received prompt wound cleansing followed by early antiviral therapy (30).

#### 3.1.3 Simian varicella virus (SVV)

##### 3.1.3.1 Etiology

SVV, officially classified as *Cercopithecine alphaherpesvirus 9,* is a neurotropic alphaherpesvirus belonging to the family *Herpesviridae*, subfamily *Alphaherpesvirinae*, and genus *Varicellovirus*. SVV is an enveloped double-stranded DNA virus with a genome of approximately 124.1 kb, and its genomic structure and organization are similar to those of human varicella-zoster virus (VZV). SVV and VZV share approximately 70–75% genomic homology, with homologous sequences located at corresponding positions in their genomes, resulting in a largely colinear genomic organization (42). SVV serves as the primary etiologic agent of naturally occurring varicella-like illness in NHPs. This disease manifests clinical and pathological features that closely mirror those observed in human VZV infections. No standardized serotype or genotype classification has been established for SVV.

##### 3.1.3.2 Host

SVV and VZV have distinct host ranges, and cross-species disease has not been reported. Experimental inoculation of VZV fails to induce varicella-like symptoms in NHPs; conversely, natural SVV infection has not been reported in humans (43). SVV primarily infects Old World monkeys, and outbreaks have been reported in patas, African green, pig-tailed, Japanese, and cynomolgus macaques. Because VZV exhibits strict host specificity and cannot be readily studied in conventional *in vivo* models, SVV infection in rhesus macaques is commonly used to study VZV pathogenesis, latency, and reactivation (44).

##### 3.1.3.3 Clinical signs and pathogenesis

SVV infection is characterized by systemic symptoms including fever, malaise, and a widespread vesicular skin rash, often accompanied by visceral dissemination. Most affected animals recover within 2 weeks, but SVV outbreaks can cause substantial morbidity and mortality, with severe cases developing pneumonia or hepatitis (45). Following the acute phase of infection, the virus establishes lifelong latency in neural ganglia, similar to VZV infection in humans (43). In the experimental rhesus macaque model, the course of infection has been well characterized following intrabronchial inoculation. Cutaneous varicella typically develops between 7 and 10 dpi, with skin lesions peaking at approximately 14 dpi. The lesions then gradually resolve, with complete clinical resolution by 21 dpi (44).

##### 3.1.3.4 Transmission route

SVV is highly contagious and can spread rapidly among susceptible NHP populations, similar to VZV transmission in humans. Epidemiological and experimental evidence suggests that SVV is transmitted predominantly via aerosols, with the respiratory epithelium and conjunctival surfaces serving as major routes of entry. Direct contact with vesicular fluid may also contribute to transmission and the spread of SVV within NHP facilities (44).

##### 3.1.3.5 Epidemiology

Historically, SVV was first identified in the mid-1960s within a colony of African green vervet monkeys, and since then, sporadic epizootics have been documented in zoos and NHP research facilities worldwide (46). SVV outbreaks can occur unpredictably and spread rapidly in captive environments. The restricted host range of SVV may reflect long-term coevolution with its primate hosts, similar to the host-specific relationship between VZV and humans (45). Facility-level serological surveys have reported SVV seropositivity of approximately 20% in pig-tailed macaques and 14.55–30.95% in cynomolgus macaques at selected breeding facilities (47, 48).

##### 3.1.3.6 Diagnostics, vaccine and therapeutics

SVV infection is diagnosed based on clinical features, particularly fever and vesicular rash, together with laboratory confirmation by direct viral detection methods. During acute infection, PCR-based assays can be performed using vesicular lesion swabs, scabs, skin biopsies, whole blood, or PBMCs to detect SVV genomic sequences (44). Serological assays detecting SVV-specific antibodies may support diagnosis by identifying previous exposure or seroconversion and useful for surveillance or outbreak investigations (49). Antiviral agents, including acyclovir, phosphonoacetic acid, FIAU, and BV-ara-U, have been evaluated for the treatment of SVV infection, with early studies reporting reduced mortality and control of clinical disease. At present, there is no licensed SVV-specific vaccine for NHPs. VZV-based vaccination has provided partial protection against SVV challenge in experimental studies, but vaccination is not currently a standard quarantine measure for macaque colonies (50).

#### 3.1.4 Simian cytomegalovirus (CMV)

##### 3.1.4.1 Etiology

CMVs are betaherpesviruses with large, linear double-stranded DNA genomes that have co-evolved with their hosts. CMVs exhibit strong host specificity and retain the conserved genomic organization typical of cytomegaloviruses (51). No standardized serotype or genotype classification has been established for simian cytomegaloviruses. Instead, they are generally distinguished by host species and strain or isolate designations, such as rhesus cytomegalovirus (RhCMV) or cynomolgus macaque cytomegalovirus (CyCMV). Among simian CMVs, RhCMV is the best-characterized model for human CMV (HCMV) (52, 53). The RhCMV genome contains approximately 230 predicted open reading frames (ORFs), of which 138 (60%) encode proteins homologous to HCMV proteins (54). The RhCMV genome is also colinear with those of other betaherpesviruses and shares extensive coding homology with HCMV, making RhCMV a useful model for HCMV research (51).

##### 3.1.4.2 Host

Primate cytomegaloviruses have been identified in a wide range of primate hosts, including humans, great apes such as chimpanzees, bonobos, gorillas, and orangutans, Old World monkeys such as rhesus and cynomolgus macaques, African green monkeys, and baboons, and New World monkeys such as owl monkeys and squirrel monkeys (55). Cytomegaloviruses are thought to have co-evolved with their host species for more than 80 million years, resulting in strong host specificity and host-adapted genetic characteristics (56). HCMV and NHP CMVs are generally host-restricted, and natural transmission between humans and NHPs has not been clearly documented (57). However, a rhesus macaque-derived CMV has been experimentally shown to infect cynomolgus macaques, indicating that transmission between closely related macaque species is possible (58).

##### 3.1.4.3 Clinical signs

RhCMV infection is typically persistent and asymptomatic in healthy rhesus macaques. However, under immunosuppressive conditions or during co-infections such as SIV, viral reactivation may lead to clinically apparent disease, including interstitial pneumonia, gastroenteritis, lymphadenitis, and other end-organ lesions (53). RhCMV-associated immune responses have also been linked to hypophysitis and endocrine dysfunction in SIV-infected rhesus macaques (59). CyCMV is a host-associated cytomegalovirus of cynomolgus macaques and establishes persistent infection. Clinical disease is uncommon and occurs primarily in immunosuppressed animals (60, 61).

##### 3.1.4.4 Transmission route

For RhCMV, horizontal transmission occurs primarily through virus shed in breast milk, saliva, and urine from infected animals. Seropositive animals may continue to shed the virus intermittently or persistently throughout life. Although less common, transplacental transmission has also been reported as a possible route of infection (52, 62). One study measured CMV viral loads in urine, saliva, and plasma from SIV-negative rhesus macaques with postnatal CMV infection across different age groups. Among 1-year-old macaques, only one of eight animals shed virus in saliva. In contrast, all seven 2-year-old macaques and all nine 3-year-old macaques shed CMV in both urine and saliva. Among 17 adult macaques, CMV was detected in 71% of urine samples and 65% of saliva samples; however, the viral loads were lower than those in the 2 and 3-year-old groups (62).

##### 3.1.4.5 Epidemiology

A seroprevalence study of 231 captive macaques at the German Primate Center reported that more than 90% of adult animals were seropositive for CMV, with seroprevalence increasing with age (63). In seven wild NHP species from Taï National Park, CMVs showed host-associated lineage diversity, with a high prevalence in some species (64).

##### 3.1.4.6 Diagnostics, vaccine and therapeutics

Serological surveillance for macaque CMVs is commonly performed using ELISA-based assays to detect CMV-specific IgG antibodies. qPCR assays are also used with various specimens, including whole blood, plasma, urine, saliva, and lymphoid tissues, to detect CMV DNA and assess viral shedding or tissue-associated infection (65). Because CMVs exhibit strong species specificity, molecular assays should be selected or designed for the CMV species associated with each host. In a rhesus macaque model of congenital RhCMV infection, maternal RhCMV DNAemia peaks early after infection, whereas viral shedding in saliva and urine begins within several weeks and may persist (66). Because CMV may not always be detected in blood, testing urine, saliva, or tissue specimens in addition to blood may increase the likelihood of detection.

No licensed CMV vaccine is currently available. CMV has a large and complex genome, high strain diversity, and multiple immune evasion mechanisms, which complicate vaccine development (67). Naturally acquired immunity to CMV does not completely prevent reinfection, reactivation, or congenital transmission. In NHP organ transplantation models, antiviral agents such as valganciclovir and ganciclovir have been used for CMV treatment or prophylaxis. However, their efficacy may be limited in severely immunosuppressed animals, and recurrence or clinical disease has been reported after discontinuation of therapy (68, 69).

### 3.2 Simian retroviruses

Retroviruses are enveloped viruses containing two copies of a positive-sense single-stranded RNA genome within a viral capsid. Replication involves reverse transcription and integration of proviral DNA into the host genome. Major retroviruses of concern in NHPs include simian immunodeficiency virus (SIV), simian type D retrovirus (SRV), and simian T-cell lymphotropic/leukemia virus (STLV).

#### 3.2.1 Simian immunodeficiency virus (SIV)

##### 3.2.1.1 Etiology

SIV are lentiviruses belonging to the family *Retroviridae*, together with human immunodeficiency viruses (HIVs). More than 40 SIV lineages have been identified in natural and non-natural hosts, primarily among wild NHP species in Africa. Because SIVs can cross species barriers, they are considered viruses of zoonotic concern. SIV genomes exhibit high mutation rates due to the error-prone nature of reverse transcriptase, allowing rapid genetic variation and adaptation (70). Despite high levels of viral replication, SIVs in their natural hosts generally cause little or no disease, reflecting long-term adaptation between the virus and host. In contrast, experimental SIV infection of non-natural hosts, such as macaques, can cause persistent immune activation, progressive immunodeficiency, and AIDS-like disease (71).

##### 3.2.1.2 Host

African NHPs, including African green monkeys, sooty mangabeys, mandrills, and chimpanzees, serve as natural hosts for diverse SIV lineages. Cross-species transmission of SIVs from African primates to humans gave rise to HIV-1 and HIV-2 (72). Natural hosts may maintain chronic SIV infection with high viral loads without developing overt disease. In contrast, humans and macaques are not natural hosts for SIV lineages and have not undergone the same long-term virus–host adaptation. In macaques, infection with pathogenic SIV strains can result in progressive immunodeficiency and AIDS-like disease (71, 73).

##### 3.2.1.3 Clinical signs and pathogenesis

Following transmission to a new host, SIV infection may result in a wide range of outcomes, ranging from asymptomatic infection to progressive disease (74, 75). In natural hosts, such as African green monkeys and sooty mangabeys, SIV infection is generally non-progressive despite high levels of viral replication, and infected animals rarely develop AIDS-like disease. However, this pattern is not universal among natural hosts. In wild chimpanzees, simian immunodeficiency virus of chimpanzee (SIVcpz) infection has been associated with increased mortality, reduced fertility, progressive CD4 + T-cell loss, and AIDS-like immunopathology (76–78).

In macaques, pathogenic SIV infection is characterized by an AIDS-like syndrome with progressive immunodeficiency and multiple clinical manifestations. Commonly reported clinical signs include lymphadenopathy, diarrhea, weight loss, wasting syndrome, lethargy, anorexia, pneumonia, dermatitis or rash, chronic enteritis, neurologic impairment, and opportunistic infections, which may ultimately lead to death in severe cases. Comparative studies in rhesus macaques have also shown that clinical outcomes can vary depending on host origin and viral strain (79, 80). SIV infection or seroconversion has rarely been documented in humans, primarily following occupational exposure. However, there is no clear evidence of persistent infection or disease progression in these cases, and the pathogenicity of SIV in humans remains uncertain (81).

##### 3.2.1.4 Transmission route

In natural hosts, SIV transmission appears to occur predominantly through sexual contact following sexual maturity, although the relative contribution of different transmission routes varies by host species and ecological context (82). In wild grivet monkeys, SIV seropositivity increases with age and sexual activity, consistent with sexual transmission as an important route (83). In contrast, mother-to-infant transmission is relatively rare in natural hosts such as sooty mangabeys, possibly because infants have fewer target cells susceptible to SIV infection (84).

In macaque models, SIV transmission has been demonstrated experimentally through several routes, including intravenous, intrarectal, intravaginal, oral, and breast-feeding transmission. Mucosal challenge models are particularly useful for studying sexual and perinatal transmission relevant to HIV infection (85, 86). Nontraumatic oral exposure to cell-free SIV can result in infection in adult macaques, and breast-feeding transmission has been demonstrated in rhesus macaque mother–infant pairs (84, 86, 87).

The most plausible routes of SIV transmission to humans include hunting, butchering, and handling meat from infected NHPs, which may result in direct contact with infected blood or tissues. Laboratory exposure to SIV-infected macaque blood or tissues have also been reported, indicating a potential risk of transmission through occupational exposure (81, 88).

##### 3.2.1.5 Epidemiology

In natural hosts, SIV prevalence varies substantially by host species, geographic region, age, and sex. Serological analyses of diverse NHP species have shown considerable differences among species, with some having no detectable infection, whereas others, such as mandrills, have shown seropositivity rates as high as 52%. A molecular epidemiological study of 225 free-ranging African green monkeys in South Africa also reported a high prevalence of SIV. Seropositivity was generally higher in adult females than in males and lower in younger animals (89, 90). SIV infection in chimpanzees is of particular interest because SIVcpz is closely related to HIV-1. A study of wild chimpanzees in southern Cameroon using nucleic acid and antibody testing reported an SIVcpz prevalence of approximately 29–35% (91).

Although macaques are widely used as experimental hosts in SIV research, simian immunodeficiency virus of macaque (SIVmac) is thought to originated through cross-species transmission of SIV from sooty mangabeys in U.S. primate centers (75). In a serological survey of 39 free-ranging rhesus macaques in Nepal, all animals tested negative for SIV by ELISA. Similarly, a survey of 1,229 captive rhesus macaques from two research colonies found no seropositive animals using ELISA and confirmatory assays (92).

##### 3.2.1.6 Diagnostics, vaccine and therapeutics

In SPF rhesus macaques, serological assays are commonly used for SIV diagnosis because SIV-specific antibodies develop following infection. These assays include enzyme immunoassays, multiplexed liquid microbead or slide array assays, and Western blot immunoassays. However, when Western blot results are indeterminate or inconclusive, PCR testing can be used for confirmation (93). In SIV-infected macaques, disease progression can be monitored by quantifying plasma viral load using qPCR (94). In sooty mangabeys, SIV-specific antibodies and viral RNA can also be detected non-invasively in feces and urine, and these measurements correlate with plasma viral load (95).

SIV vaccination and therapeutic strategies have been studied in macaque models, which are commonly used for preclinical evaluation of HIV vaccines and treatments (96–98). SIV vaccine platforms evaluated in macaques include live-attenuated SIV, poxvirus vectors, adenovirus/Modified Vaccinia Ankara (MVA) prime–boost regimens, adenovirus/protein vaccines, and RhCMV/SIV vectors. Vaccine efficacy is typically evaluated using repeated mucosal SIV challenge, plasma viral load, CD4 + T-cell preservation, SIV-specific antibody responses and T-cell responses, and tissue reservoir measurements (99–102).

Therapeutic studies in SIV-infected macaques use suppressive antiretroviral therapy (ART) regimens to model HIV treatment and reservoir persistence. ART may be combined with therapeutic vaccination, TLR7 agonist, or broadly neutralizing antibodies, followed by analytical treatment interruption to evaluate viral rebound and post-ART control (103–106). In natural hosts, SIV research focuses primarily on non-progressive infection, control of immune activation, and host adaptation rather than vaccination (70, 77, 107, 108).

#### 3.2.2 Simian type D retrovirus (SRV)

##### 3.2.2.1 Etiology

SRVs are enveloped, single-stranded RNA viruses classified within the genus *Betaretrovirus* of the family *Retroviridae* and show considerable genetic and antigenic diversity. Traditionally, they have been classified into serotypes SRV-1 to SRV-5, with additional divergent genotypes, including SRV-6 and SRV-7, identified in macaque populations (109). Sequence analysis of the *env* gene has revealed genetic variation within SRV-2, with multiple subtypes reported in different macaque species (110).

##### 3.2.2.2 Host

SRVs primarily infect Asian macaques of the genus *Macaca*, including rhesus macaques, cynomolgus macaques, pig-tailed macaques, Japanese macaques, and Celebes black macaques (110). Although SRVs are important pathogens in macaque colonies, natural transmission to humans has rarely been reported. Among the recognized serotypes, SRV-1 is most commonly associated with rhesus macaques, whereas SRV-2 is more frequently detected in cynomolgus and pig-tailed macaques (13).

##### 3.2.2.3 Clinical signs and pathogenesis

Unlike HIV or SIV, SRV is not restricted to CD4 + T cells and can infect several types of immune cells, including T cells, B cells, and macrophages (111). Many SRV-infected macaques are asymptomatic carriers and may shed virus without clinical signs. However, some animals develop acute or chronic immunodeficiency. Clinical signs may include weight loss, often exceeding 10% of body weight, chronic diarrhea, fever, and lymphadenopathy (112). Infected animals are also more susceptible to opportunistic infections caused by parasites, bacteria, or fungi (111). SRV infection has been associated with noma, a severe form of necrotizing gingivitis or stomatitis, and SRV-2 infection has also been associated with proliferative lesions such as retroperitoneal fibromatosis (110). SRV infection can affect the interpretation of studies using macaques because of its effects on immune function, hematologic parameters, and susceptibility to opportunistic infections. This is particularly relevant to studies involving vaccines, transplantation, immunology, infectious disease, and immunotoxicology (112).

##### 3.2.2.4 Transmission route

SRV is shed in various body fluids, including plasma, saliva, urine, and feces (113). Horizontal transmission is thought to occur primarily through close direct contact between infected and susceptible macaques, with exposure to body fluids during biting, scratching, fighting, grooming, or other social interactions (114). Indirect transmission may also occur through contaminated fomites, food or water exposed to saliva, urine, or feces (115). Vertical transmission may occur transplacentally, during birth, or through breastfeeding from an infected mother to her offspring (116).

##### 3.2.2.5 Epidemiology

SRV infection is enzootic in some Asian macaque populations of the genus *Macaca*, but prevalence varies widely among captive colonies. Reported rates range from 0% in SRV-free colonies to approximately 3–5% in some endemic colonies, while prevalence may exceed 50% in affected colonies (117–119). In one laboratory-bred cynomolgus macaque colony, 397 of 419 animals (95%) were positive for SRV-specific antibodies or viral RNA in plasma (120).

##### 3.2.2.6 Diagnostics, vaccine and therapeutics

Diagnosis of SRV infection is typically based on a combination of serologic and molecular assays. Serologic screening, such as ELISA or immunoblot-based antibody testing, is commonly used for colony surveillance (11). Broad-range or generic qPCR assays have been developed to detect multiple SRV serotypes (121). Experimental vaccines, including inactivated and recombinant viral vector vaccines, have shown protective effects in experimental studies, but no licensed vaccine is currently available (122, 123). No specific antiviral treatment for SRV infection is currently available.

#### 3.2.3 Simian T cell lymphotropic/leukemia virus (STLV)

##### 3.2.3.1 Etiology

STLVs are enveloped, positive-sense, single-stranded RNA retroviruses belonging to the family *Retroviridae* and genus *Deltaretrovirus*. STLVs are the simian counterparts of human T-lymphotropic viruses (HTLVs) and are grouped within the primate T-lymphotropic viruses (PTLVs). Three major PTLV types have been described, comprising HTLV-1/STLV-1, HTLV-2/STLV-2, and HTLV-3/STLV-3 (124). Among them, STLV-1 is the best-characterized and is closely related to HTLV-1, sharing approximately 90–95% sequence identity or amino acid similarity, depending on the viral region analyzed (125). HTLV-1 and STLV-1 strains often cluster phylogenetically by geographic origin rather than host species, consistent with repeated cross-species transmission between NHPs and humans (126).

##### 3.2.3.2 Host

The natural hosts of STLVs are NHPs, particularly Old World monkeys and apes from Africa and Asia, including macaques, baboons, African green monkeys, guenons, mangabeys, mandrills, chimpanzees, and gorillas (127, 128). The host range and prevalence vary by primate species, geographic region, and viral lineage (124). Human infection with simian-origin PTLVs has been reported. Phylogenetic and epidemiological studies have documented repeated zoonotic transmission of STLV-1 from NHPs to humans, especially in Central Africa, where exposure to NHP blood and tissues can occur through hunting, bushmeat handling, and bites (129).

##### 3.2.3.3 Clinical signs and pathogenesis

Most STLV-1-infected NHPs remain clinically asymptomatic (130). However, STLV-1 has been associated with adult T-cell leukemia/lymphoma (ATL)-like T-cell leukemia/lymphoma and other lymphoproliferative diseases in naturally infected NHPs (131). Reported manifestations include lymphadenopathy and systemic lymphoid or visceral involvement. In contrast, inflammatory conditions such as uveitis are well documented in human HTLV-1 infection but have not been clearly associated with STLV infection in NHPs. A characteristic hematologic finding in ATL-like disease is the presence of atypical lymphocytes with multilobulated nuclei, commonly referred to as “flower cells” (131). In contrast, PTLV-2 and PTLV-3 have not been conclusively associated with specific diseases in either humans or NHPs. HTLV-2 has been sporadically associated with mild neurological or HAM/TSP-like disorders, whereas HTLV-3/STLV-3 infections have mainly been described in asymptomatic individuals and molecular epidemiological studies, without a clearly established disease association (124). Zoonotic transmission is best documented for STLV-1, whereas human infections related to STLV-3 have been reported only rarely.

##### 3.2.3.4 Transmission route

STLV-1 is transmitted primarily through horizontal routes, particularly during aggressive interactions such as bites and fighting. Sexual transmission has also been reported in some primate species. Vertical transmission from mother to offspring has been reported, although it appears to be less common and may occur through breastfeeding (130, 132). The transmission routes of STLV-2 and STLV-3 are less well characterized than those of STLV-1.

##### 3.2.3.5 Epidemiology

The prevalence of STLV infection varies by viral type, primate species, geographic origin, and housing conditions. STLV-1 is broadly distributed among Old World monkeys and apes in Africa and Asia, whereas STLV-2 has been reported mainly in bonobos and STLV-3 in several African NHP species (132–134). STLV-1 is highly prevalent in Japanese macaques; one study detected antibodies in 320 of 533 animals (60%), and subsequent studies reported prevalence exceeding 60%. In the same study, only 1 of 163 rhesus macaques was seropositive (131). A high seroprevalence was also reported in captive sooty mangabeys, with 22 of 39 animals (56%) testing positive (135). Lower prevalence has been reported in some captive macaque colonies. A survey of 848 captive macaques detected STLV-1 antibodies in 27 animals (3.2%), while a study of 337 captive cynomolgus macaques reported STLV-1 single infection in 4% and SRV-2/STLV-1 coinfection in 9% of animals (136).

##### 3.2.3.6 Diagnostics, vaccine and therapeutics

ELISA is commonly used as a first-line serologic screening method to detect antibodies against PTLV/STLV antigens, often followed by confirmatory assays such as Western blot or line immunoassay (137). PCR based assays can detect STLV proviral DNA in peripheral blood mononuclear cells or tissues, including samples from clinically asymptomatic animals (131). Because serologic assays may yield indeterminate or cross-reactive results and do not directly demonstrate proviral infection, PCR and sequence analysis can be used together with serology to confirm infection and determine viral type (138). Although experimental vaccine strategies have been investigated mainly for HTLV-1 and STLV-1 models, no commercially available vaccine or specific antiviral therapy currently exists for STLV infection (139).

### 3.3 Simian polyomavirus

#### 3.3.1 Simian virus 40 (SV40)

##### 3.3.1.1 Etiology

SV40 is a non-enveloped, icosahedral, double-stranded DNA virus belonging to the family *Polyomaviridae* and genus *Betapolyomavirus*. It is closely related to the human polyomaviruses JC virus (JCV) and BK virus (BKV) (140–142). SV40 is not generally classified into serotypes. Instead, genetically distinct strains and variants have been identified in macaques, including isolates with archetypal or rearranged regulatory regions (143–146). These variants may differ in their replication properties and pathogenicity.

##### 3.3.1.2 Host

SV40 is a naturally occurring polyomavirus of Asian macaques, with rhesus macaques recognized as a major natural host; cynomolgus macaques are also susceptible to infection (147). Human exposure to SV40 has been associated primarily with the administration of poliovirus vaccines contaminated with SV40 between 1955 and 1963 (146). Although zoonotic transmission of SV40 remains controversial, SV40 seroreactivity has been reported in zoo personnel with close contact with NHPs, suggesting the possibility of occupational exposure (148–150).

##### 3.3.1.3 Clinical signs and pathogenesis

In healthy macaques, SV40 infection is usually asymptomatic or persistent. However, in immunocompromised animals, SV40 can cause clinically significant disease, including tubulointerstitial nephritis, meningoencephalitis, and progressive multifocal leukoencephalopathy (PML)-like demyelinating disease (145, 151). These lesions are associated with productive SV40 infection in susceptible tissues, particularly the kidney and brain. SV40 large T antigen can also interfere with cellular tumor suppressor pathways and is responsible for the transforming properties of SV40 observed in experimental systems (152). In NHPs, the kidney is considered a major site of SV40 persistence, particularly in renal tubular epithelial cells, and viral reactivation may result in shedding in urine (153). In humans, SV40 exposure occurred historically through contaminated poliovirus vaccines and has also been investigated in relation to occupational contact with NHPs; however, direct zoonotic transmission and its clinical significance remain uncertain.

##### 3.3.1.4 Transmission route

In NHPs, SV40 is commonly shed in urine, and natural transmission is thought to occur mainly through direct or indirect exposure to infectious excreta or contaminated materials, particularly via fecal-oral or urine–oral routes. Respiratory transmission has also been proposed based on experimental intranasal infection and detection of the virus in the upper respiratory tract, although its role in natural transmission remains unclear (141, 146). Vertical transmission of SV40 has not been established as a major route in NHPs. Seroconversion data from a cynomolgus macaque breeding colony suggested that transmission was neither vertical nor perinatal (154).

##### 3.3.1.5 Epidemiology

SV40 seroprevalence is high in several macaque species and may reach 95%, depending on age and geographic origin (144, 147). In most animals, the virus persists without clinical signs, and clinical disease is uncommon. However, in immunocompromised macaques, SV40 may cause diseases affecting the central nervous system, kidneys, and lungs. Central nervous system lesions in affected macaques may resemble those of progressive PML in humans (155, 156).

##### 3.3.1.6 Diagnostics, vaccine and therapeutics

Diagnosis of SV40 infection in NHPs is based on serological and molecular assays (111). Serological tests, including ELISA and virus-like particle-based immunoassays, are used to detect antibodies indicative of prior exposure, whereas PCR-based assays are used to detect SV40 DNA in blood, urine, or tissues samples, particularly from the kidney and central nervous system (145, 150). No vaccines or specific antiviral treatments is currently available for SV40. Therefore, prevention in NHPs facilities relies on appropriate management of immunocompromised animals, control of environmental contamination by excreta, and measures to reduce occupational exposure among personnel.

#### 3.3.2 Measles virus

##### 3.3.2.1 Etiology

Measles virus is an enveloped, single-stranded, negative-sense RNA virus of the family *Paramyxoviridae* and the genus *Morbillivirus* and is highly transmissible (157, 158). Viral particles range from spherical to pleomorphic in shape and measure approximately 120–270 nm in diameter (159). Measles virus infections in NHPs are caused by the same viral serotype that infects humans, and no NHP-specific serotype has been identified. Wild-type measles viruses are classified into 8 clades and 24 genotypes based primarily on H and N gene sequences (160).

##### 3.3.2.2 Host

Although humans are the only natural reservoir of measles virus, many NHP species, including apes and both Old World and New World monkeys, are susceptible to infection. Spontaneous measles outbreaks have been reported in zoos, sanctuaries, and primate research or breeding facilities, often following exposure to infected humans (155, 161, 162).

##### 3.3.2.3 Clinical signs and pathogenesis

Disease severity varies by species, age, immune status, and husbandry conditions, with clinical outcomes ranging from subclinical infection to severe or fatal disease. In NHPs, clinical signs appear after an incubation period of approximately 1–2 weeks and resemble those observed in humans. Initial signs include cough, fever, coryza, conjunctivitis, facial erythema and edema, and malaise, followed by a maculopapular rash that primarily affects the face and trunk. Abortion may occur in pregnant animals. Measles can also cause lymphopenia and suppression of both humoral and cell-mediated immune responses, which may persist after the acute phase of infection (25, 163, 164).

##### 3.3.2.4 Transmission route

Measles virus is transmitted primarily through respiratory droplets and aerosols. Because humans are the only natural reservoir, naturally occurring measles infections in NHPs are generally considered anthroponotic (157). When measles virus is introduced into zoos, sanctuaries, or primate research and breeding facilities, it can spread among susceptible NHPs via respiratory droplets and aerosols. However, because infection induces long-lasting protective immunity, the number of susceptible animal’s declines rapidly, limiting sustained transmission within NHP populations (165, 166). Vertical transmission has not been clearly demonstrated as an important route of measles virus transmission in NHPs, although infection during pregnancy may result in abortion or other reproductive complications.

##### 3.3.2.5 Epidemiology

Measles virus infection in NHPs is predominantly anthroponotic, with transmission occurring primarily from infected humans to susceptible NHPs. Because measles can spread rapidly in susceptible NHP colonies and may cause substantial morbidity and mortality, particularly in certain species or immunologically naïve captive populations, outbreaks can seriously affect colony health. Mortality rates in macaque measles outbreaks have been reported to range from approximately 5 to 30%, varying according to species, age, immune status, and facility condition (162). In 1986–1987, a large measles outbreak occurred at the California Primate Research Center, with 147 recognized cases among macaques and an mortality rate of approximately 28% (167). A natural measles outbreak in Japanese macaques at a Korean zoo resulted in approximately 21% mortality (168). Serological evidence of measles virus exposure has also been reported in asymptomatic macaques, indicating that clinical observation alone may not identify all infected animals (161). Laboratory testing is therefore important for detecting infection and controlling measles outbreaks in NHP colonies.

##### 3.3.2.6 Diagnostics, vaccine and therapeutics

Measles antibody testing in NHPs includes IgG ELISA to assess prior exposure or vaccine-induced immunity, IgM ELISA or paired-serum IgG seroconversion to identify recent infection, and neutralization assays to measure protective antibody responses (169, 170). Because serology does not directly demonstrate viral shedding, RT-PCR detection of measles viral RNA in respiratory samples can be used together with serology when acute infection is suspected (171). The live attenuated measles vaccine developed for humans can also be used in NHPs to prevent and control measles outbreaks (165, 172). Maternal antibodies should be considered when determining the timing of vaccination. Vaccination after 6 months of age has been reported to be effective in rhesus macaques (169). However, vaccination may interfere with tuberculin skin test results and is not recommended for animals with active infection, immunosuppression, or pregnancy (172).

#### 3.3.3 Additional viral diseases of clinical and quarantine concern in macaques

Other viruses relevant to clinical and quarantine concern include filoviruses such as Marburg virus and Ebola virus, monkeypox virus (MPXV), and rabies virus. Testing for these viruses may be required depending on the current epidemiological situation, clinical signs, and country-specific import or quarantine requirements.

##### 3.3.3.1 Etiology

Ebola virus and Marburg virus are enveloped, single-stranded, negative-sense RNA viruses belonging to the family *Filoviridae* and the genera *Orthoebolavirus* and *Orthomarburgvirus*, respectively (173). Among orthoebolaviruses, Reston virus (RESTV) is of particular concern for macaque colonies because natural outbreaks have occurred in cynomolgus macaques (174). Rabies virus is also an enveloped, single-stranded, negative-sense RNA virus and belongs to the genus *Lyssavirus* within the family *Rhabdoviridae* (175). In contrast, MPXV is an enveloped, double-stranded DNA virus belonging to the genus *Orthopoxvirus* within the family *Poxviridae* (176). These viruses are classified using virus-specific taxonomic and phylogenetic schemes rather than a common serotype-based classification.

##### 3.3.3.2 Host

All four viruses can infect humans and may cause severe or fatal disease. Several NHP species, including macaques, are susceptible to infection with these viruses. Natural Ebola virus infections have been reported in gorillas and chimpanzees, while African green monkeys imported from Uganda were the source of human infection during the first recognized Marburg virus outbreak in 1967 (177, 178). RESTV is particularly relevant to macaques because repeated outbreaks have occurred in captive cynomolgus macaque colonies (179). Natural monkeypox virus infection has been documented in African NHPs, including wild sooty mangabeys, and macaques are susceptible to experimental infection (180, 181). Rabies virus infection has been reported in a variety of NHP species, including macaques, with marmoset-associated rabies being particularly well documented in Brazil (182).

##### 3.3.3.3 Clinical signs and pathogenesis

In macaques, pathogenic filoviruses such as Marburg virus and Ebola virus cause acute systemic disease characterized by fever, anorexia, lethargy, petechiae, and, in severe cases, hemorrhage, shock, multiorgan failure, and death. Experimental infection of cynomolgus and rhesus macaques can result in rapidly progressive and frequently fatal disease, depending on the virus, dose, and route of inoculation (183). In humans, case fatality rates range from 24 to 88% for Marburg virus disease and from 25 to 90% for Ebola disease (184). MPXV infection in macaques can cause fever, anorexia, lethargy, lymphadenopathy, and characteristic cutaneous lesions that progress from macules and papules to vesicles, pustules, and crusts. Severe infection may also involve the respiratory tract. Disease severity and mortality vary considerably with the viral strain, dose, and route of inoculation (185). Rabies virus causes progressive neurologic disease, with clinical signs including abnormal behavior, ataxia, hypersalivation, paralysis, seizures, and coma. Naturally occurring rabies is uncommon in macaques, and a species-specific case fatality rate has not been established (182). However, clinical rabies is generally fatal once neurologic signs develop (182).

##### 3.3.3.4 Transmission route

Marburg and Ebola viruses are mainly transmitted through direct contact with infected blood or other body fluids through broken skin or mucous membranes (186). Although information on natural transmission among NHPs is limited, parenteral, mucosal, and aerosol exposure can result in Marburg virus infection in experimental NHP models (187). Ebola virus infection has also been induced experimentally in macaques following intramuscular, oral, conjunctival, and aerosol exposure (188, 189). MPXV is transmitted mainly through close contact with infectious skin or mucosal lesions, body fluids, or contaminated materials. Respiratory exposure may also contribute to transmission, although it appears to be less common. Animal-to-human transmission may occur through bites, scratches, or direct contact with infected animals (190). In NHP experimental models, MPXV infection has been established through aerosol, intravenous, intradermal, and subcutaneous routes (185). Rabies virus is transmitted through infected saliva, most commonly by bites and less commonly by scratches or contact of saliva with broken skin or mucous membranes (191).

##### 3.3.3.5 Epidemiology

Macaques are not considered established reservoir hosts for these viruses, and naturally occurring infections in macaque populations have generally been sporadic or poorly documented. The natural reservoir of Marburg virus is the Egyptian fruit bat (*Rousettus aegyptiacus*), whereas African green monkeys are susceptible incidental hosts and were associated with the first recognized Marburg virus outbreak in 1967 following importation from Uganda (192). Gorillas and chimpanzees in Central and West Africa can develop severe and fatal Ebola virus infection, and natural outbreaks in these wild African great apes have resulted in substantial mortality (177). RESTV is particularly relevant to macaques because outbreaks have repeatedly occurred in captive cynomolgus macaques imported from or bred in the Philippines (179). The natural reservoir of MPXV has not been clearly identified, although African rodents are considered the most likely reservoir hosts. Natural infection has been documented in African NHPs, including sooty mangabeys, whereas naturally acquired infection in macaques appears to be uncommon; macaques are instead widely used as experimental models of MPXV infection (180, 185). Rabies has been reported sporadically in various NHPs, including macaques, marmosets, and capuchin monkeys; common marmosets are particularly relevant in parts of Brazil, where a distinct rabies virus transmission cycle involving this species has been described (182).

##### 3.3.3.6 Diagnostics, vaccine and therapeutics

Laboratory diagnosis relies mainly on molecular detection methods, including PCR or RT-PCR, with antigen and serological assays used where appropriate. Similar molecular methods are used in NHPs, whereas rabies is generally confirmed postmortem by detection of viral antigen or RNA in brain tissue. Licensed vaccines are available for the prevention of Ebola virus disease, MPXV infection, and rabies in humans, whereas no licensed vaccine is currently available for Marburg virus disease (193). No vaccines are specifically licensed for routine use against these diseases in NHPs, although macaques are widely used in experimental vaccine studies (194). Monoclonal antibody therapies are available for Ebola virus disease, whereas treatment of Marburg virus disease remains supportive (193). Treatment options for MPXV infection are limited, and the clinical efficacy of tecovirimat has not been established (195). No effective treatment is available once clinical signs of rabies develop (196). No specific therapeutics are approved for routine treatment of these infections in NHPs. Experimental therapeutic candidates have been evaluated for some of these viral infections.

## 4 Discussion

Recent WHO pathogen prioritization efforts have emphasized family-level preparedness and response strategies (197). This concept is also relevant to NHP quarantine because many viruses of concern in primate colonies, particularly members of the families *Retroviridae* and *Herpesviridae*, are capable of establishing persistent or latent infections (Table 2). In addition, other important pathogens, such as polyomaviruses and mycobacteria, may persist chronically after introduction. These biological features make complete elimination difficult. Moreover, NHP breeding and research facilities often rely on long-term group-housing systems, which may facilitate pathogen maintenance and transmission within colonies. Given that major NHP-associated viruses can be transmitted through horizontal and, in some cases, vertical routes, the introduction of infected animals may increase the likelihood of transmission to other animals within the colony (Table 3). Therefore, family-level diagnostic preparedness, continuous surveillance, and internationally coordinated quarantine practices are important for NHP facilities.

**Table 2:** Quarantine-associated viruses in non-human primates and their etiological characteristics.

Virus	Etiology			Variants	
	Family	Genus	Related human pathogens	Serotype	Genotype or lineages
B virus	Herpesviridae	Simplexvirus	Human herpes simplex virus	ND	Macaque host specific genotype
Simian varicella virus	Herpesviridae	Varicellovirus	Human varicella-zoster virus	ND	ND
Simian cytomegalovirus	Herpesviridae	Cytomegalovirus	Human cytomegalovirus (Closely realted RhCMV)	ND	Host species-specific CMV species
Simian immunodeficiency virus	Retroviridae	Lentivirus	Human immunodeficiency virus	ND	Host species-specific SIV lineages
Simian type D retrovirus	Retroviridae	Betaretrovirus	ND	ND	SRV6 and SRV7
Simian T cell lymphotropic/ leukemia virus	Retroviridae	Deltaretrovirus	Human T lymphoytopic disease	ND	HTLV-1/STLV-1, HTLV-2/STLV-2 HTLV-3/STLV-3
Simian virus 40	Polyomaviridae	Betapolyomavirus	human polyomaviruses JC virus (JCV) and BK virus (BKV)	ND	Classical and novel SV40 types
Measles virus	Paramyxoviridae	Morbillivirus	Same virus as in humans	Serotype 1	24 genotypes, 8 clades (A-H)
Ebola virus	Filoviridae	Orthoebolavirus	Same virus as in humans	ND	Multiple lineages
Marburg virus	Filoviridae	Orthomarburgvirus	Same virus as in humans	ND	Multiple lineages
Rabies virus	Rhabdoviridae	Lyssavirus	Same virus as in humans	Serotype 1	Multiple lineages
Monkeypox virus	Poxviridae	Orthopoxvirus	Same virus as in humans	ND	Multiple lineages 4 clades

ND, Not determined.

**Table 3:** Transmission and control measures for quarantine-associated viruses in non-human primates.

Virus	Transmission in NHPs		Interspecies transmission		Intervention	
	Horizontal	Vertical	Human to NHPs	NHPs to human	Vaccine	Therapeutics
B virus	Direct contact (biting etc.), fomite	ND	ND	Yes	No	Yes
Simian varicella virus	Respiratory	ND	ND	ND	Yes	Yes
Simian cytomegalovirus	Oral (breast milk), direct (saliva and urine)	Yes	ND	ND	No	Yes
Simian immunodeficiency virus	Sexual activity, Oral (breast feeding)	Yes (breast feeding)	ND	Yes	Yes (research only)	Yes (research only)
Simian type D retrovirus	Direct contact (biting etc.)	Yes (breast feeding, placental)	ND	ND	Yes (research only)	No
Simian T cell lymphotropic/ leukemia virus	Direct contact (biting etc.)	Yes	ND	Yes	No	No
Simian virus 40	Direct contact (urine), respiratory	No	ND	ND	No	No
Measles virus	Respiratory	ND	Yes	Yes	Yes	No
Ebola virus	Direct contact (blood etc.)	Yes	ND	Yes	Yes (human)	Yes (human)
Marburg virus	Direct contact (blood etc.)	ND	ND	Yes	No	No
Rabies virus	Direct contact (bite etc.)	ND	ND	Yes	Yes (human)	No
Monkeypox virus	Direct (skin lesions etc.) respiratory, fomite	Yes	ND	ND	Yes (human)	Limited

ND, Not determined.

These considerations highlight the importance of active microbiological monitoring because many NHP-associated viruses generally cause asymptomatic or only mild infections, although various clinical manifestations may occasionally occur (Figure 2). Nevertheless, microbiological surveillance standards differ among countries, and enforceable requirements remain limited. Given that NHPs are commonly distributed from a limited number of breeding facilities to research institutions worldwide, effective quarantine should be addressed not only at the level of individual facilities but also harmonized international standards and coordinated surveillance (18). From a One Health perspective, there is a need to define a minimum set of diseases for quarantine screening, particularly latent or persistent viral infections and tuberculosis, to reduce the risks associated with the international movement of NHPs. Their potential for subclinical infection and zoonotic transmission further highlights the importance of integrated surveillance for NHP colony health, occupational safety, research reliability, and public health (Figure 3). However, important knowledge gaps remain regarding transmission dynamics in NHP research and facility settings and the associated risks to humans. The current evidence base is also limited by uneven species coverage, heterogeneity among institutions and surveillance practices, and insufficient data for certain viral pathogens. Given these uncertainties, a precautionary approach based on a minimum level of routine surveillance is warranted to reduce the risk of undetected infection and transmission. Considering the variability in screening practices among institutions and the limitations of direct antigen or pathogen detection for many retroviruses and herpesviruses, we propose that this minimum surveillance framework should include at least annual antibody testing for major persistent or latent viruses, including SIV, SRV, STLV, B virus, SCMV, and SVV.

**Figure 2:**
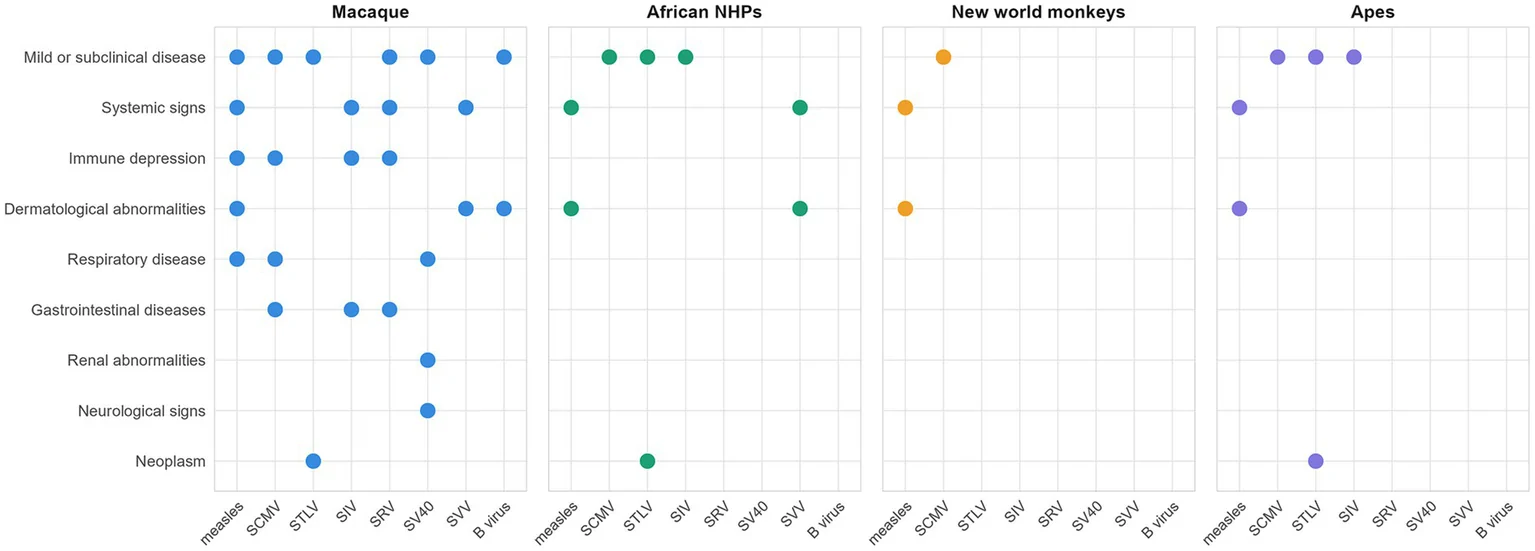
Differential diagnostic overview of quarantine-associated major viral diseases in non-human primates by clinical presentation and host group. Dots indicating commonly reported clinical manifestations of each viral disease in the corresponding NHP host group. Clinical categories including mild or subclinical disease, systemic signs, immune depression, dermatological abnormalities, respiratory disease, gastrointestinal disease, renal abnormalities, neurological signs, and neoplasia. Host groups comprising macaques, African NHPs, New World monkeys, and apes. SCMV, simian cytomegalovirus; STLV, simian T-cell lymphotropic/leukemia virus; SIV, simian immunodeficiency virus; SRV, simian type D retrovirus; SV40, simian virus 40; SVV, simian varicella virus. SCMV, simian cytomegalovirus; STLV, simian T-lymphotropic virus; SIV, simian immunodeficiency virus; SRV, simian retrovirus/simian betaretrovirus; SV40, simian virus 40; SVV, simian varicella virus.

**Figure 3:**
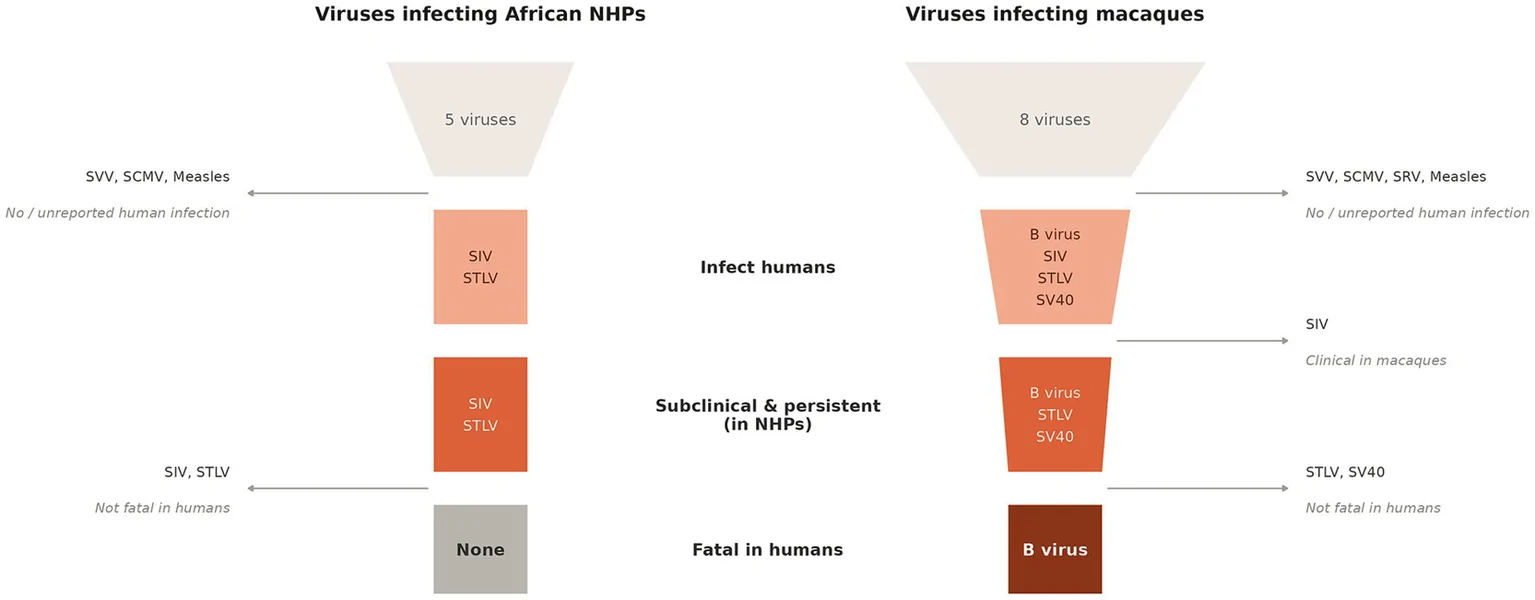
Zoonotic potential and clinical significance of major quarantine-associated viruses in non-human primates and humans. Comparison of major viruses infecting African NHPs and macaques according to human infectivity, subclinical and persistent infection in NHPs, and fatality in humans. SIV, simian immunodeficiency virus; STLV, simian T-cell lymphotropic virus; SVV, simian varicella virus; SCMV, simian cytomegalovirus; SV40, simian virus 40.

In addition to determining seropositivity using established diagnostic cut-off values, longitudinal evaluation of antibody titers, rather than reliance on a single time-point measurement, may be important for assessing infection status and identifying changes associated with exposure, viral reactivation, or potential transmission within a colony. Antibody results should also be evaluated beyond the individual animal level, with comparisons among groups, housing rooms, and facilities to identify temporal changes in exposure and transmission patterns. Nevertheless, antibody testing should not be used in isolation. Serological findings should be interpreted together with clinical observation, necropsy findings, antigen or molecular assays, hematological and serum biochemical data, and expert interpretation. Because infected animals may exhibit only mild or no apparent clinical signs and viral shedding may be intermittent, repeated antigen or molecular testing of animals with suspected infection or positive serological findings may be necessary to confirm active viral shedding.

Furthermore, facility-level standard operating procedures (SOPs) should be established in advance for each pathogen to define appropriate response measures according to different combinations of diagnostic results, such as seropositivity alone or concurrent positive serological and antigen or molecular findings. The detection of these pathogens should prompt the implementation of appropriate SOPs to prevent further transmission within the colony, restrict the transfer of infected or exposed animals, and protect personnel from zoonotic exposure. For high-risk zoonotic agents such as B virus, rapid post-exposure evaluation and appropriate antiviral prophylaxis or treatment are particularly important to reduce the risk of severe human disease. Thus, SOPs, routine training, exposure-response plans, and access to appropriate medical countermeasures should be established before incidents occur.

In addition to viruses of the families *Retroviridae* and *Herpesviridae*, four viral diseases of particular concern were also included because of their high pathogenicity in humans and recent public health importance. Although NHPs are not generally considered natural reservoirs for these viruses, they may become infected as susceptible or incidental hosts and therefore warrant consideration during quarantine. Because these infections can cause recognizable clinical signs in macaques, as in humans, careful clinical surveillance is important during the quarantine period. Unlike persistent or latent viral infections, quarantine surveillance for Marburg virus, Ebola virus, MPXV, and rabies virus should focus on the early recognition of suggestive clinical signs, followed by the implementation of appropriate precautionary biosafety measures before prompt diagnostic testing using antigen or molecular assays and, when necessary, postmortem examination. Rapid identification of suspected or confirmed cases should then trigger appropriate public health and facility-level response measures, including assessment and management of potentially exposed personnel, isolation of affected animals, and measures to prevent further transmission.
